# Functional Training for Lower Extremities in Stroke Survivors: A Scoping Review

**DOI:** 10.7759/cureus.58087

**Published:** 2024-04-11

**Authors:** Meenakshi Jharbade, Sivakumar Ramachandran, Shankar V, John Solomon M

**Affiliations:** 1 Department of Physiotherapy, Sri Ramachandra Institute of Higher Education and Research. (Deemed to be University), Chennai, IND; 2 Department of Neurology, Sri Ramachandra Institute of Higher Education and Research. (Deemed to be University), Chennai, IND; 3 Department of Physiotherapy, Manipal College of Health Professions, Manipal Academy of Higher Education, Manipal, IND

**Keywords:** rehabilitation, task oriented training, functional training, hemiplegia, stroke

## Abstract

Engaging in meaningful and repetitive goal-oriented functional tasks can effectively enhance neuroplasticity and facilitate recovery following a stroke. This particular approach has primarily been studied in relation to functional outcomes and has predominantly focused on late subacute and chronic stroke patients. However, there is a lack of information regarding the standardized protocol of lower extremity functional training, its constituent elements, and its impact on motor recovery during the early subacute phase of stroke. The aim of this study was to examine the available evidence related to the intervention protocol of lower extremity functional training in order to identify common training elements and assess their impact on motor and functional outcomes in stroke survivors. A systematic search was conducted on PubMed and Scopus, covering the period from 2000 to 2022. A total of 1786 articles were retrieved and screened based on predefined inclusion criteria. A total of 36 articles were included in this review. The primary findings were classified into categories such as intervention protocols for functional training and their constituent elements, outcome measures utilized, minimal clinically important differences (MCID) reported, and the conclusions drawn by the respective studies. Only a limited quantity of studies reported on the intervention protocol of lower extremity functional training. The majority of these studies focused on the efficacy of functional training for enhancing gait and balance, as evaluated through functional outcome assessments, particularly in the context of chronic stroke patients. In most studies, the evaluation of outcomes was typically based on statistical significance rather than clinical significance. In light of these findings, it is recommended that future studies be conducted during the early subacute phase of stroke to further investigate the impact of functional training on motor outcomes. This will contribute to a broader understanding of the benefits of functional training in facilitating motor recovery in the lower extremities and its clinical significance in stroke survivors.

## Introduction and background

Stroke is a significant contributor to disability leading to functional and motor deficits [1]. Impairments in motor function of the lower limb following a stroke are prevalent and can exhibit varying degrees of severity and presentation, contingent upon the location and size of the lesion. Post-stroke individuals commonly endure a range of disabilities and impairments that significantly impact their postural stability, mobility functions, and activities of daily living [2]. Commonly implemented rehabilitation interventions to augment recovery in stroke patients include task-specific training, robot-assisted training, constraint-induced movement therapy, body weight-supported treadmill training, balance training, gait training, functional electrical stimulation, mirror therapy, and virtual reality [3,4].

Functional training is a therapy approach centered around the task-oriented practice of goal-directed functional tasks. These functional tasks emphasize the engagement of muscle groups working in unison, rather than isolated muscle movements [5]. Functional training includes purposeful, repetitive, intensive mass practice of meaningful functional activities to the patient. Engagement in functional training within an enriched environment aids in enhancing neural plasticity and facilitating motor recovery [4]. The impact of functional training on functional abilities like gait, balance, and sit-to-stand has been extensively investigated [6,7]. While motor recovery is a crucial aspect, evidence suggests that functional training has been extensively examined in terms of functional recovery rather than motor recovery in stroke survivors [8,9]. Studies indicate that there exists a distinction between functional recovery and motor recovery and these distinctions are measurable with functional and motor outcomes [10,11].

Functional training is administered in various forms within stroke rehabilitation such as repetitive functional task practice, repetitive task practice [12], task-related training [13], task-oriented therapy [14], and circuit class training [15]. The intervention protocol by Dean et al. has been utilized as a reference in some studies [16-18]. However, the documentation of standard intervention protocol for lower extremity functional training remains unclear in the existing literature. The majority of the studies on functional training have been conducted on chronic and late subacute stroke populations [19]. There is a dearth of research on the early subacute stroke population regarding lower extremity functional training, although Liu et al. suggest that implementing interventional strategies within two weeks of the subacute stroke phase during early rehabilitation may lead to improved outcomes [20].

The concept of minimal clinically important difference (MCID) is employed to denote the smallest minimal change that signifies an improvement in the patient’s perspective. It is worth noting that while treatment effects can be identified through statistically significant differences, it is not always the case that statistical difference corresponds to practical or clinical significance. Outcomes defined using MCID rather than statistical significance can capture changes in outcomes that are clinically relevant and practical in the context of health care treatments [21]. In the realm of functional training, few studies have taken into account the MCID when explaining the outcomes [17,22-25].

The aim of this scoping review was to address several questions. Specifically, we sought to determine whether functional training has been studied in the early subacute, late subacute, and chronic stroke populations. Additionally, we aimed to explore whether there exists a standardized protocol for lower extremity functional training as well as whether there are common exercises present in the training. Furthermore, we aimed to assess the outcomes of the studies focused on functional or motor recovery of the lower extremity and whether these outcomes were evaluated in terms of MCID or solely based on statistical significance. 

## Review

Methodology

The Arksey and O’Malley scoping review methodology framework [26], as well as the Preferred Reporting Items for Systematic Reviews and Meta-Analyses (PRISMA) extension for scoping reviews guidelines, were employed for the reporting of scoping review in this study.

The scoping review research questions are as follows: (i) Is there a standardized protocol or common elements in lower extremity functional training, (ii) Has functional training been studied in all stages of stroke, (iii) What are the common functional and motor outcomes used to assess the effect of lower extremity functional training in stroke survivors, and (iv) Are the reported outcomes based on clinically meaningful changes (MCID) or solely on statistical significance.

Identifying Relevant Studies

To identify relevant studies, we formulated a search strategy and conducted searches in two electronic databases PubMed and Scopus. We used keywords such as "Stroke", "Cerebrovascular Accident", "Hemiplegia", "Task-oriented training", "Task-specific training", "Functional training", "Circuit class training", "Rehabilitation", " Conventional Physiotherapy", "Walking", "Gait", "Balance", "Postural control", "Sit to stand" and "Weight bearing". The search was limited to articles published between 2000 and 2022. Boolean operators and medical subject headings (MeSH) terms related to these keywords were used in the search.

Study Selection

For the study selection process, we applied the following inclusion criteria: articles published in the English language, study participants as stroke survivors, intervention as functional training for lower extremity, outcomes studied for functional and motor recovery, and full-text articles of randomized controlled trials. Two independent reviewers (MJ, SR) conducted an initial screening based on the title and abstract of the records. After removing duplicates, the full text of the selected articles was read. Any conflicts in the screening and study selection process were resolved by a third reviewer (SV/JS). The data was managed using the Rayyan application reference manager [27] and Microsoft Word (Microsoft Corporation, Redmond, Washington, United States).

Charting the Data

The data from the articles was extracted, including the author, year of publication, days post-stroke, stages of stroke, sample size, intervention received by the control and experimental groups, intervention duration, outcome measures, and study conclusions.

Collating, Summarizing, and Reporting the Results

After discussing and interpreting the data, the final results were summarized into different categories. The results were reported in subcategories based on the stages of stroke studied, whether the functional training implemented had a standard protocol or common elements, the functional and motor outcome measures, and the reported outcomes for clinical and statistical significance.

Results

**Figure 1:**
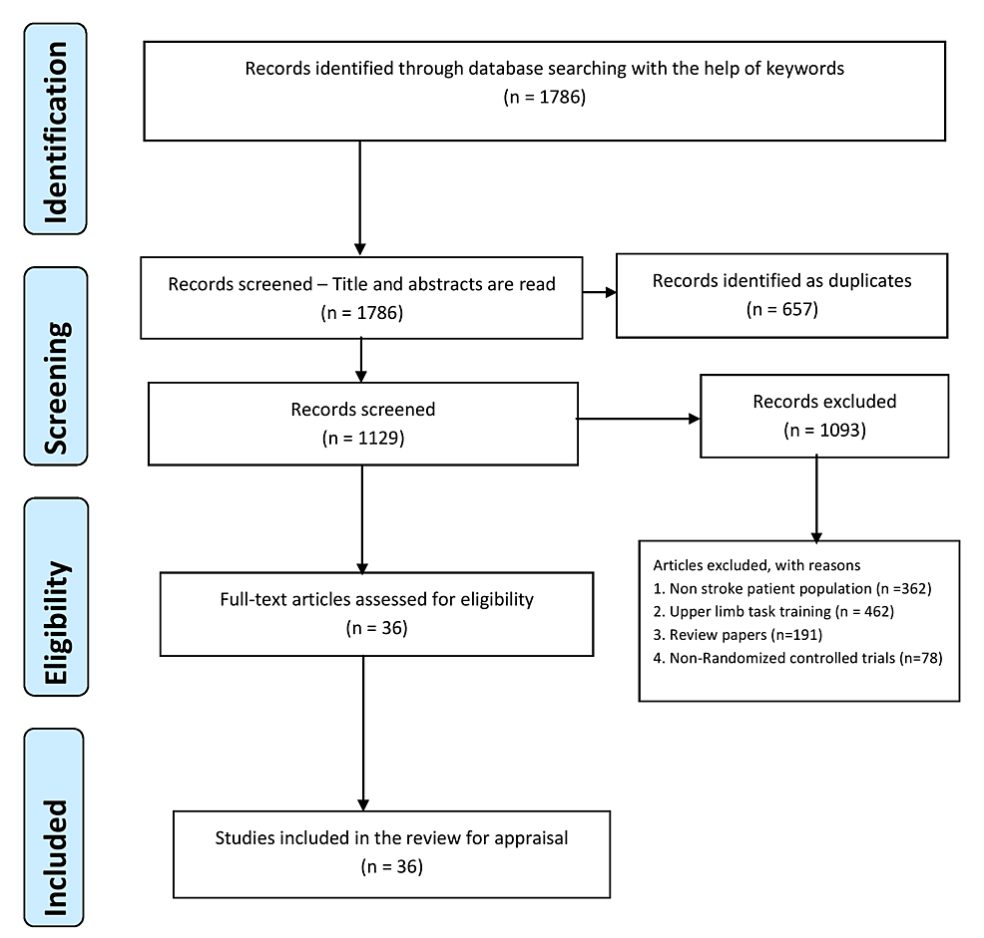
PRISMA flow diagram of the study selection process PRISMA: Preferred Reporting Items for Systematic Reviews and Meta-Analyses

A total of 1786 articles were obtained from the databases of which 657 duplicate articles were eliminated. The title and abstract of 1129 articles were assessed. After the initial assessment, 1093 articles were excluded. Based on the inclusion criteria, 36 full-text articles were read and included in the review. The process of selecting studies for the review paper is presented in Figure 1, using the PRISMA flow diagram. 

Intervention Protocol and its Components for Lower Extremity Functional Training

The intervention protocol and its components for functional training of the lower extremities were described in nine studies involving stroke patients. These studies include those by Salbach et al. [18], Mendoza et al. [22], Kuberan et al. [23], Choi and Kang [28], Mudge et al. [29], English et al. [30], Dean et al. [31], Ma et al. [32], and Martins et al. [33]. The various components of lower extremity functional training that were investigated include walking in 29 studies, balance in 23 studies, sit-to-stand in 13 studies, stair climbing in six studies, and weight bearing in three studies.

Walking was trained in different forms such as walking on various surfaces, comfortable and fast walking, obstacle course walking, Indoor and outdoor walking, carrying objects while walking, tandem walking, backward walking, and walking on a treadmill [18,22,23,28-31]. Stepping exercises involved the use of blocks and stair climbing [18,28,30-32]. Activities in the standing position included standing with feet close together, in a tandem position, standing on one leg with support, standing within parallel bars, reaching tasks, and marching in place [28-31]. Activities in a sitting position included reaching tasks and sitting on a Swiss ball [23,30,31]. Sit-to-stand practice was performed using chairs of varying heights [22,23,29-31,33]. The exercises were progressed gradually by increasing the number of repetitions or the difficulty of the tasks.

However, it was observed that some studies did not provide detailed information about their intervention protocols, and in many cases, the specific functional tasks used were not clearly documented among the studies [17,34,35].

Functional Training in Various Stages of Stroke

The categorization of studies followed the guidelines set by the stroke recovery and rehabilitation roundtable on timelines of post-stroke, which classified them into early subacute (seven days to three months post stroke), late subacute (three to six months post stroke), and chronic (after six months post stroke) stages [36]. Out of the 36 studies included in this review, the majority (21 studies) focused on participants in the chronic stage of stroke [18,22,23,28,29,31-33,35,37-48]. There were six studies that focused on the late subacute phase [24,25,49-52] and eight studies that focused on the early subacute phase [16,17,30,34,53-56]. One study did not specify the stage of stroke [57].

Intervention Duration

The duration of functional training interventions varied across studies with three weeks in three studies [23,34,41], four weeks in 17 studies [16,17,22,24,28-32,39,40,42,45-47,53,56], six weeks in eight studies [18,35,43,44,49,54,55,57], eight weeks in two studies [37,52], 10 weeks in three studies [38,48,50] and 12 weeks in three studies [25,33,51].

Functional and Motor Outcomes

The commonly used functional outcome measures were the Berg Balance Scale in 20 studies [16-18,24,28,29,30,34,37,38,40-42,44,46,48,49,51,53,57], Timed Up and Go test in 18 studies [18,23-25,31,37,39,41,42,44,46-49,51,55-57], Six-Minute Walk test in 16 studies [16-18,22,24,25,29,31,33,44,47,49,51,52,55,56], 10-Metre Walk test in 13 studies [17,22,29,31,33,39,41,42,46,49,51,52,55], Step test in six studies [29,31,41,47,48,56], kinematic analysis for gait and sit to stand in five studies [31,43,45,47,54], Dynamic Gait Index in four studies [23,37,38,49], Functional Ambulation Category in four studies [24,25,52,55], Functional Reach test in four studies [17,35,49,50], Fall Efficacy Scale in four studies [23,25,42,55], Activities Specific Balance Confidence Scale in three studies [29,40,48], Trunk Impairment Scale in two studies [46,50], Stroke Impact Scale in two studies [25,55], Modified Stair Climb test in two studies [25,55] and Modified Barthel Index in two studies [16,28]. The motor outcome used in the included studies was the Fugl Meyer assessment scale of motor recovery after stroke, reported in three studies [16,34,37].

Clinically Meaningful Changes (MCID)

Eight studies provided information on the clinically meaningful changes in the outcome measures [17,22-25,30,51,55]. These studies used MCID to interpret the results for the Six-Minute Walk test in five studies [17,24,25,51,55], 10-Metre Walk test in four studies [17,22,51,55], Berg Balance Scale in two studies [30,51], Stroke Impact Scale [55], Dynamic Gait Index [23], Timed Up and Go test [51], Two-Minute Walk test [30], and Five-Metre Walk test [30].

The study characteristics for the 36 included studies in the scoping review can be found in Table 1. 

**Table 1:** Summary of 36 studies included in the scoping review investigated for lower extremity functional training in stroke survivors FMA: Fugl Meyer assessment scale of motor recovery after stroke; BBS: Berg Balance scale; 6MWT: 6-Minute Walk test; 10 MWT: 10-Metre Walk test; TUG: Timed Up and Go test; SIS: Stroke Impact Scale; FAC: Functional Ambulation Category; DGI: Dynamic Gait Index; FES: Fall Efficacy Scale; FRT: Functional Reach test; MBI: Modified Barthel Index; ABC scale: Activities Specific Balance Confidence scale; TEMPA: Test d'Evaluation des Membres Suprieurs de Personnes Agres for upper extremity; SSQOL: Stroke Specific Quality of Life Scale; TOT: task-oriented training; TST: task-specific training; CCT: circuit class training; DTT: dual-task training; VRT: virtual reality training; PNF: proprioceptive neuromuscular facilitation; ADL: activities of daily living; N: total sample size; E: experimental group; C: control group; CPT: conventional physiotherapy; PT: physiotherapy; COP: centre of pressure; MCID: minimal clinically Important difference.

Author and year of publication	Post stroke duration (Early subacute/Late subacute/chronic stroke phase)	Sample size (N)	Intervention for Experimental and Control group	Duration of Intervention (weeks)	Outcome measures	Conclusion of the study
Kim et al., 2016 [16]	30 days post stroke. (Early subacute)	N - 20 (E – 10 and C – 10)	E – Circuit training, C – Conventional Physiotherapy (CPT)	4 weeks	Fugl Meyer Assessment scale of motor recovery, Berg Balance Scale, 6-Minute Walk Test, Modified Barthel Index.	Circuit training was effective to improve the motor control of paretic lower extremity, balance and walking endurance in early subacute phase of stroke.
Outermans et al., 2010 [17]	E – 22 days, C – 23 days post stroke (Early subacute)	N – 44 (E – 23 and C – 21)	E – usual physiotherapy and High intensity circuit class training, C – usual physiotherapy (PT)	4 weeks	6 Minute Walk Test, 10-Metre Walk Test, Berg Balance Scale, Functional Reach Test.	Circuit training was effective to improve the gait speed and walking endurance in early subacute stroke patients. Clinically significant improvements were noted in 6 MWT and 10 MWT considering the MCID values.
Salbach et al., 2004 [18]	E – 239 days, C – 217 days post stroke (Chronic)	N – 91 (E – 44 and C – 47)	E – Task-Oriented Training, C – Functional tasks training for upper limb. (Intervention protocol reported)	6 weeks	Berg Balance Scale, Timed Up and Go Test, 6-Minute Walk Test, Five-Metre Walk Test.	The study concluded that task-oriented training was effective in improving walking distance and speed in chronic stroke patients.
Mendoza et al., 2021 [22]	E – 3 years, C – 5 years Post stroke (chronic)	N – 18 (E – 9 and C – 9)	Task group – Task oriented circuit class training, Impairment group – Circuit class training focused on specific impairments. (Intervention protocol reported)	4 weeks	6-Minute Walk Test, 10-Metre Walk Test, Timed Up-and-Down Stairs.	The study suggested that circuit class therapy can improve walking abilities in chronic stroke patients. Fast gait velocity reached to MCID Values in task group.
Kuberan et al., 2017 [23]	11 months post stroke (chronic)	N – 26 (E – 13 and C – 13)	E – Task-oriented training, C – Conventional physiotherapy (Intervention protocol reported)	3 weeks	Dynamic Gait Index, Timed Up-and-Go Test, Fall Efficacy Scale.	Task oriented exercises with altered sensory input was found to be effective in improving dynamic balance and functional mobility and reduction in fear of fall. Clinically meaningful changes (MCID) was found in DGI scores in the experimental group.
Kim et al., 2017 [24]	E – 90 days, C – 120 days. (Late subacute)	N – 30 (E – 15 and C – 150)	E – Task oriented circuit training, C – Conventional physiotherapy	4 weeks	Berg Balance Scale, Timed Up-and-Go test, 6-Minute Walk Test, Functional Ambulation Classification.	The Study concluded that task oriented circuit training improve the gait in subacute stroke patients. Clinically meaningful changes were noted in 6MWT in task oriented training group.
Ingrid et al., 2012 [25]	E – 91 days, C – 103 days post stroke (late subacute)	N – 242 (E –125 and C –117)	E – Task oriented circuit training, C – usual Physiotherapy	12 weeks	Stroke Impact Scale, Rivermead Mobility Index, Falls Efficacy Scale, Nottingham Extended Activities of Daily Living scale, Hospital Anxiety and Depression Scale, Fatigue Severity Scale, Motricity Index, 6-Minute Walk Test, 5-Metre Walking Speed Test, Functional Ambulation Category, Timed Up-and-Go Test, Timed Balance Test, Modified Stairs Test, and Letter Cancellation Task.	Task oriented circuit training was effective to improve gait abilities in late subacute phase of stroke. Circuit training showed clinically meaningful change of scores in 6MWT.
Choi et al., 2015 [28]	More than 180 days post stroke (Chronic)	N – 20 (E – 10 and C – 10)	E – Task oriented Training, C – Traditional rehabilitation therapy (Intervention protocol reported)	4 weeks	Berg Balance Scale, Modified Barthel Index, Self Efficacy Scale.	Task oriented training was effective to improve the balance, ADL, self efficacy in chronic stroke patients.
Mudge et al., 2009 [29]	E – 3.3 years, C – 5.8 years post stroke. (Chronic)	N – 58 (E – 31 and C – 27)	E – Circuit class therapy, C – sessions by occupational therapist and consisted of 4 social and 4 educational sessions. (Intervention protocol reported)	4 weeks	Step Watch Activity Monitor, 10-Meter walk test, 6-Minute Walk Test, Activities Specific Balance Confidence Scale, Berg Balance Scale, Rivermead Mobility Index.	Circuit training showed improvements in gait endurance in chronic stroke patients.
English et al., 2007 [30]	E – 29 days, C – 24 days post stroke. (Early subacute)	N – 68 (E – 37 and C – 31)	E – Circuit class Therapy for mobility and Upper limb function. C – Usual PT (Intervention protocol reported)	4 weeks	Five-Meter Walk Test, Two-Minute Walk Test, Berg Balance Scale, Motor Assessment Scale - upper limb.	The study demonstrated the feasibility and safety of circuit class therapy received by inpatients with early subacute stroke phase. Circuit training was effective in improving greater degree of independence in walking at hospital discharge of patients. Outcomes were checked for clinically meaningful changes.
Dean et al., 2000 [31]	E – 2 years, C – 1 year post stroke. (Chronic)	N – 9 (E – 5 and C – 4)	E – Task oriented circuit class training, C – Upper limb task training. (Intervention protocol reported)	4 weeks	6-Minute Walk Test, Timed Up-and-Go test, 10-Metre Walk Test, Step Test, Laboratory Measures for Sit to Stand and Walking.	The study concluded that task oriented circuit class training improved the walking speed and endurance, weight bearing on paretic lower extremity, force production with paretic lower extremity during sit to stand and ability to balance on paretic lower extremity while stepping in chronic stroke patients. The study also proved that the improvements with circuit class training in experimental group were maintained for two months after the discontinuation of training.
Ma et al., 2017 [32]	E – 30 months, C – 32 months post stroke (Chronic)	N – 30 (E – 15 and C – 15)	E – Task oriented training, C – simple repetitive balance activity in a balance board and trampoline (Intervention protocol reported)	4 weeks	MTD Balance system	Task oriented activities was effective in improving weight bearing, static standing balance and gait.
Martin et al., 2020 [33]	6 months post stroke (Chronic)	N – 36 (E – 18 and C – 18)	E – task specific circuit training for UL+LL, C – Global stretching, memory exercises, education session (Intervention protocol reported)	12 weeks	Human Activity Profile, 10-Metre Walk Test, TEMPA – UL, 6-Minute Walk Test, Dynamometers, Stroke Specific Quality of Life Scale.	Task specific circuit training had improved the quality of life in chronic stroke patients.
Rose et al., 2011 [34]	10 days post stroke. (Early subacute)	N – 180 (E – 72 and C – 108)	E – Task oriented Circuit training. C – standard physical therapy	3 weeks	Five-Meter Walk Test, Berg Balance Scale, Functional Independence Measure, Fugl-Meyer Assessment Scale of Motor Recovery After Stroke.	Circuit training had improve the gait speed in early subacute stroke patients. This feasibility study has showed the successful implementation of circuit training model to stroke inpatients with 10 days post stroke duration.
McClellan et al., 2004 [35]	E – 6.5 months, C – 4.5 months post stroke. (Chronic)	N – 23 (E – 13 and C – 10)	E – Home based task oriented mobility training and challenging activities for balance during standing and walking, C – Upper limb exercises (Carr and Shepherd 1987)	6 weeks	Functional Reach Test, Motor Assessment Scale – walking, Sickness Impact Profile – QOL.	The study concluded that 6 weeks of home based task oriented program improved the balance and mobility in chronic stroke patients.
Malik et al., 2021 [37]	3-6 month post stroke (Chronic)	N – 52 (E – 26 and C – 26)	E – Task oriented training (TOT), C – Task oriented training and Virtual reality training (VRT)	8 weeks	Fugl Meyer Assessment scale (Lower Extremity), Timed Up and Go Test, Berg Balance Scale, Dynamic Gait Index.	VRT combined with TOT improved the physical performance, balance and mobility outcomes better in comparison to TOT alone in chronic stroke patients.
Anandan et al., 2020 [38]	6 months post stroke (Chronic)	N – 74 (E – 37 and C – 37)	E – Task specific training (TST) C= PNF therapy	10 weeks	Modified Ashworth Scale, Action Reach Arm Scale, Berg Balance Scale, Dynamic Gait Index.	Task specific therapy was effective to improve the balance and gait in chronic stroke patients.
Iqbal et al., 2020 [39]	6 month post stroke (Chronic)	N – 64 (E – 32 and C – 32)	E – Dual Task training (DTT), C – Conventional physiotherapy (CPT)	4 weeks	Timed Up and Go Test, 10-Metre Walk Test, Cadence, Step Length, Stride Length, Gait Cycle Time.	Dual task training showed significant improvements in all spatial and temporal gait variables in chronic stroke patients.
Park MH et al., 2017 [40]	16 months post stroke (Chronic)	N – 26 (E – 14 and C – 12)	E – Task oriented training, C – General physical therapy	4 weeks	Berg Balance Scale, Korean Activities-Specific Balance Confidence Scale.	Task oriented training with altered somatosensory inputs can improve balance in chronic stroke patients.
Park KT et al., 2016 [41]	6 months post stroke (Chronic)	N –12 (E – 6 and C – 6)	E – Circuit training, C – Indoor walking training	3 weeks	Smart Step Test, Berg Balance scale, 10-Meter Walk Test, Timed Up-and-Go test.	Circuit training program designed with obstacle course was effective to improve the walking speed and balance in chronic stroke patients.
Park GD et al., 2016 [42]	6 months post stroke (Chronic)	N – 40 (E – 20 and C – 20)	E – weight shift training and Multidirectional stepping task training, C – General physical therapy	4 weeks	Berg Balance Scale, Timed Up-and-Go test, 10-Meter Walk Test, Falls Efficacy Scale.	Repetitive stepping task exercises in diverse directions was effective to improve the balance, gait ability, and falls efficacy in chronic stroke patients.
Choi et al., 2017 [43]	More than 180 days post stroke. (chronic)	N – 36 (E – 18 and C – 18)	E – Stair task training, C – Weight support and balance training.	6 weeks	Kinematic Motion Analysis to measure gait.	Significant differences were found between groups in swing phase time. Stair task training was found to be effective in improving gait after stroke.
Kim et al., 2016 [44]	More than 180 days post stroke. (Chronic)	N – 23 (E – 12 and C – 11)	E – Group task-oriented circuit training (GTCT with 2-3 patients in a group). C = Individual task-oriented circuit training (ITCT) group	6 weeks	Berg Balance Scale, Timed Up-and-Go Test, 6-Minute Walk Test.	Group task-oriented circuit training improved the balance and walking in chronic stroke patients.
Song et al., 2015 [45]	Group 1 – 37 months, Group 2 – 31 months, Group 3 – 28 months post stroke (Chronic)	N – 30 (C1 – 10, E2 – 10 and E3 – 10)	C 1 – Conventional PT, E 2 – Conventional PT and Individual-based task-oriented circuit training, E 3 – Conventional PT and Group-based task-oriented circuit class training.	4 weeks	Kinematic Analysis of gait, Two-Minute Walking Test.	Task-oriented circuit training improved the gait ability in chronic stroke patients.
Kim et al., 2012 [46]	E – 7 years, C – 13 years post stroke. (Chronic)	N – 20 (E – 10 and C – 10)	E – Task-oriented training, C – Conventional physical therapy.	4 weeks	Trunk Impairment Scale, Berg Balance Scale, Timed Up-and-Go Test, 10-Metre Walk Test.	Task-oriented training improved the trunk control and gait in chronic stroke patients.
Yang et al., 2006 [47]	E – 63 months, C – 64 months post stroke. (Chronic)	N – 48 (E – 24 and C – 24)	E – Task-oriented progressive resistance strength training, C – No rehabilitation training	4 weeks	GAITRite system for spatial temporal gait parameters. Kinematic Analysis, Dynamometer, 6-Minute Walk Test, Timed Up-and-Go Test, Step Test.	Progressive resistance strength training added with task-oriented training was effective in improving muscle strength of lower extremities and functional performance in gait in chronic stroke patients.
Marigold et al., 2005 [48]	E – 3.6 years, C – 3.8 years post stroke. (Chronic)	N – 61 (E – 30 and C – 31)	E – Task-oriented training, C – stretching and weight-shifting exercises	10 weeks.	Berg Balance Scale, Activities-specific Balance Confidence Scale, Timed Up-and-Go Test, Step Reaction Time, Nottingham Health Profile.	The study concluded that community-based group task-oriented sessions were effective in reducing falls and improved the standing balance and mobility in chronic stroke patients.
Ali et al., 2020 [49]	Stroke onset less than 3 months (Late subacute)	N – 22 (E – 11 and C – 11)	E – Group-based task-oriented circuit training, C – Individual circuit training	6 weeks	Motor Assessment Scale, Time Up-and-Go Test, 10-Meter Walk Test, 6-Minute Walk Test, Functional Reach Test, Dynamic Gait Index, Modified Ashworth Scale, Berg Balance Scale.	Task-oriented circuit class training was effective in improving balance, walking ability, walking competency, and walking endurance in late subacute phase of stroke.
Khallaf et al., 2020 [50]	E – 28 days, C – 28 days post stroke (Late subacute)	N – 34 (E – 17 and C – 17)	E – Task-specific training, C – Conventional physical therapy	10 weeks.	Trunk Impairment Scale, Postural Assessment Scale, Functional Reach Test, LASER Guided Digital Goniometer.	Task-specific training was effective in improving the static and dynamic postural control and trunk ranges of motion in subacute stroke phase.
Megan et al., 2018 [51]	10 weeks post stroke (Late subacute)	N – 144 (Task group – 51, Strength group – 45 and Control group – 48)	Task group – Task-oriented circuit gait training. Strength group – strength training of lower extremities while sitting and lying position. Control group – educational session on stroke management.	12 weeks	6-Minute Walk Test, 10-Metre Walk Test, Berg Balance Scale, Timed Up-and-Go Test.	The study concluded that implementation of task-oriented circuit gait training with the help of caregiver was effective in enhancing locomotor recovery and walking competency in stroke patients. Clinically meaningful (MCID) changes of scores in the task group were noted on 6MWT, 10 MWT, BBS, and TUG.
Frimpong et al., 2014 [52]	E – 2.2 months, C – 2.4 months post stroke. (Late subacute)	N – 20 (E – 10 and C – 10)	E – Task-oriented circuit training, C – conventional therapy	8 weeks	6-Minute Walk Test, 10-Metre Walk Test, Functional Ambulation Category.	Task-oriented circuit training improved the ambulatory function in subacute stroke survivors.
Arabzadeh et al., 2018 [53]	33 days post stroke (Early subacute)	N – 20 (E – 10 and C – 10)	E – Task-oriented training, C – Conventional physiotherapy	4 weeks	Berg Balance Scale, Pedobarograph System with sensors	Balance, postural sway parameters as COP path length and COP area, and symmetry in weight bearing improved significantly after implementation of task-oriented training in early subacute stroke patients.
Kerr et al., 2017 [54]	34 days post stroke. (Early subacute)	N – 93 (CPT group – 32, CPT and MPT group – 31, FST and CPT group – 30)	E 1 – Conventional PT and Movement Performance Therapy, E 2 – Conventional PT and Functional strength training focused on repetitive progressive resistive exercise during goal-directed functional activity. C = Conventional Physiotherapy	6 weeks	Kinematic Analysis for Sit-to-Stand	Functional task training had improved the sit to stand ability in early subacute stroke patients.
Renner et al., 2016 [55]	E – 39 day post stroke, C – 32 day post stroke. (Early subacute)	N – 73 (E – 34 and C – 39)	E – Group-based circuit Class training, C – Individual task training.	6 weeks	Stroke Impact Scale, Rivermead Mobility Index, Motricity Index - leg score, Functional Ambulation Category, Fall Efficacy Scale, Hospital Anxiety and Depression Scale, Fatigue Severity Scale, 6-Minute walk test, 10-Meter walk Test, Timed Balance Test, Timed Up-and-Go Test, Chair Rise Test, Modified Stairs Climb Test.	Circuit training was found to be effective in improving the gait speed and mobility in early subacute stroke phase. Clinically meaningful changes (MCID) were noted on mobility domain of Stroke Impact scale, 6MWT and 10 MWT.
Blennerhassett et al., 2004 [56]	E – 36 days, C – 50 days post stroke. (Early subacute)	N – 30 (E – 15 and C – 15)	E – Usual PT and task-oriented circuit class training, C = Usual PT and Upper limb exercise	4 weeks	6-Minute Walk Test, Timed Up-and-Go test, Step Test.	Task-oriented circuit class training was effective in improving the walking and stepping in early subacute stroke patients.
Quratul et al., 2021 [57]	Not mentioned	N – 37 (E – 19 and C – 18)	E – Circuit training, C – Virtual reality training	6 weeks	Timed up and Go test, Berg Balance scale.	Circuit training was effective in improving the gait and balance in stroke survivors.

Discussion

In the scoping review, we investigated four distinct research inquiries. Out of the 36 studies examined in the review, only nine studies provided comprehensive information regarding the interventional protocol. The protocols employed varied across the studies. The training was administered in a structured manner, such as circuit class training or customized to target specific functions of the lower limb. Functional training encompassed a range of activities conducted in seated and standing positions, including practicing sit-to-stand and walking. Based on the findings of the review, it can be concluded that an intervention protocol for functional training is necessary to facilitate the generalization of exercises for individuals with subacute and chronic stroke respectively.

Functional training has been assessed for its effectiveness, supported by its physiological basis of influencing neuroplasticity and motor recovery. However, the majority of studies have focused on functional outcomes rather than motor recovery. While motor recovery is a crucial aspect of stroke rehabilitation, functional recovery has been the primary outcome of interest in most clinical trials involving functional training interventions. Functional abilities may also improve through compensation, which entails the adoption of alternative behavioural strategies to complete tasks or achieve goals. It is important to differentiate motor recovery from compensation [58].

In addition to sit-to-stand and stepping, mobility is an essential function of the lower extremity, which is also involved in postural control and manipulating tasks [6]. A standardized protocol can be valuable for implementing and comparing interventions that target different functions of the lower extremity. Impaired postural control often results from disrupted muscle activation in the lower limbs following a stroke. The lower limbs play a critical role in maintaining balance, and their compromised function due to post-stroke impairments can lead to instability and an increased risk of falls. Postural-related activities are pivotal in eliciting muscle contractions in the lower limbs and ensuring stability and balance [59]. Oddsson conducted a study emphasizing the connection between postural control and muscle activation in the lower limbs [60]. Dean et al. illustrated the role of lower extremities in postural control during sitting reaching tasks [61]. Electromyography (EMG) findings from the study support the consistent ability of stroke patients to elicit muscle contractions in the anterior tibial and soleus muscles in the paretic lower extremity following training in seated reaching tasks [61]. Consistent with these findings, several studies have incorporated reaching tasks in sitting and standing positions, with the anticipation of improvement in lower extremity motor control [30,31].

Manipulation or handling of objects is widely recognized as an upper extremity function. The training of functional tasks involving grasping, releasing and manipulating objects has been commonly employed in hand rehabilitation following a stroke [62]. Meanwhile, in our everyday lives, the lower extremity is also utilized for object manipulation. Activities such as using the lower limb to move footwear, retrieving objects from the floor with the lower limb and kicking or moving objects using the lower limb could be classified as manipulative tasks of the lower limb. Davies recommends tasks such as holding and moving a ball beneath the paretic lower limb in order to elicit muscle contraction [63]. A study conducted by Ramachandran et al. demonstrated that training the paretic upper limb to maintain a position on an unstable surface resulted in proximal muscle contraction of the upper limb in stroke patients during the acute phase [64]. However, it is worth noting that such activities were not observed in the functional training tasks employed in stroke rehabilitation.

Initiating rehabilitation in the early stages following a stroke may prove to be efficacious and advantageous for patients as it has the potential to enhance neuroplasticity and consequently promote recovery [20]. Clinical trials have predominantly included individuals from the chronic stroke population as study participants, in comparison to the late subacute and early subacute stroke populations [19]. There exists a necessity for clinical trials to ascertain the impact of functional training on the early subacute population, as this specific time period presents an increased likelihood of neuroplastic changes, which in turn aids in facilitating improved recovery and outcomes. The literature is lacking in substantial evidence regarding the effectiveness of lower extremity functional training on motor outcomes. Among the 36 studies included in the review, three studies have only utilized the Fugl-Meyer assessment scale of motor recovery to substantiate the outcomes of the study [16,34,37].

In certain studies, the analyses implemented when assessing the study results incorporated the concept of MCID [17,22-25,30,51,55]. MCID signifies the smallest alteration that holds clinical significance from the patient’s perspective and it may accurately reflect the efficacy of a treatment or intervention. By incorporating the MCID values into the results, researchers can gain a more comprehensive understanding of the effectiveness of lower extremity functional training following a stroke.

Strengths and Limitations of the Study 

The review presents comprehensive details on various categories such as post-stroke duration, sample size, intervention methods employed in the experimental and control groups, outcome measures, reports of MCID, and conclusions drawn from the individual studies. A limitation of this study is the absence of a quality assessment for the included studies.

## Conclusions

A diverse range of functional tasks involving the lower extremities, such as transitioning from a seated to a standing position, engaging in reaching activities while sitting or standing, bearing weight while standing, and practicing walking in various forms, has been previously integrated into functional training. Functional activities for the lower extremities can involve tasks of manipulation and dynamic control of posture while standing. To determine the effectiveness of functional training in stroke survivors, it is imperative to establish a standardized protocol. Functional training in the chronic phase of stroke has been the focus of most studies comparing its efficacy to the subacute phase. The majority of these studies have emphasized functional outcome measures to demonstrate its effectiveness. However, there is a lack of evidence regarding the efficacy of functional training on the motor outcomes of the paretic lower extremity in the early subacute phase of stroke. Future clinical trials and reviews can be conducted to contribute further to the existing evidence and explore the clinical implications of lower extremity functional training in the early subacute phase of stroke.
